# Oxymatrine Ameliorates Lupus Nephritis by Targeting the YY1-Mediated IL-6/STAT3 Axis

**DOI:** 10.3390/ijms252212260

**Published:** 2024-11-14

**Authors:** Haoxing Yuan, Zheng Peng, Honglian Li, Yuzhen Rao, Kunyu Lu, Chan Yang, Chen Cheng, Shuwen Liu

**Affiliations:** 1 Guangdong Provincial Key Laboratory of New Drug Screening, NMPA Key Laboratory of Drug Metabolism Research and Evaluation, School of Pharmaceutical Sciences, Southern Medical University, Guangzhou 510515, China; yuanhaoxing1222@163.com (H.Y.); pengzheng2014@163.com (Z.P.); lihonglian202101@163.com (H.L.); yzraohorris@gmail.com (Y.R.); lky886@smu.edu.cn (K.L.); virus6522@smu.edu.cn (C.Y.); chengchen1997@i.smu.edu.cn (C.C.); 2 State Key Laboratory of Organ Failure Research, Guangdong Provincial Institute of Nephrology, Southern Medical University, Guangzhou 510515, China; 3 Innovation Center for Medical Basic Research on Inflammation and Immune Related Diseases, Ministry of Education, Southern Medical University, Guangzhou 510515, China

**Keywords:** lupus nephritis, Yin Yang 1, inflammatory factors, oxymatrine

## Abstract

Lupus nephritis (LN) is a severe form of systemic lupus erythematosus (SLE), characterized by inflammation in the renal glomeruli and tubules. Previous research has demonstrated that dihydroartemisinin (DHA) can reduce inflammatory damage in LN mouse models. Oxymatrine, which has similar biological properties to DHA, may also provide therapeutic benefits. This study aims to investigate the effects of oxymatrine on LN using a murine model and examines its molecular mechanisms through an analysis of microarray datasets from LN patients. The analysis identified differentially expressed genes (DEGs) in renal tissues, regulated by the transcription factor Yin Yang 1 (YY1), which was found to be significantly upregulated in LN patient kidneys. The results indicate that oxymatrine targets the YY1/IL-6/STAT3 signaling pathway. In cell models simulating renal inflammation, oxymatrine reduced YY1 expression and inhibited the secretion of inflammatory factors (IFs), thereby diminishing inflammation. YY1 is crucial in modulating IFs’ secretion and contributing to LN pathogenesis. Additionally, oxymatrine’s interaction with YY1, leading to its downregulation, appears to be a key mechanism in alleviating LN symptoms. These findings support oxymatrine as a promising therapeutic agent for LN, offering new avenues for treating this autoimmune kidney disorder.

## 1. Introduction

Lupus nephritis (LN), the most prevalent chronic complication of systemic lupus erythematosus (SLE), is a leading cause of mortality in individuals with SLE [1]. The primary goals in SLE management are to reduce treatment side effects and prevent organ damage, with a strong focus on preserving renal function. Proteinuria, glomerular inflammation, and renal tubular inflammation are the hallmark pathological features of LN [2]. The current therapeutic strategy for LN focuses on using immunosuppressive agents, including tacrolimus and glucocorticoids, to attain remission. Furthermore, multi-targeted therapeutic strategies are employed to promote remission. Mycophenolate mofetil, when used in combination with glucocorticoids, is suggested to reduce mortality rates, the occurrence of infections, the progression to end-stage renal disease (ESRD), and the recurrence of the disease [3]. However, to date, no existing clinical intervention has effectively slowed LN progression without causing significant side effects, highlighting the urgent need for novel therapeutic strategies.

Emerging evidence suggests that nephritis not only triggers LN pathogenesis but also accelerates its progression. The release of multiple inflammatory factors (IFs) is a key pathological feature associated with LN [4,5,6]. Studies have demonstrated that immune complex deposition in glomerular mesangial cells (GMCs) leads to glomerular inflammation, while renal tubular epithelial cells (RTECs) mediate macrophage recruitment in the kidney, inducing a local inflammatory cascade response in LN [7,8,9]. Chronic inflammation in RTECs also contributes to tubulointerstitial inflammation, which can intensify tubulointerstitial injury and ultimately lead to ESRD or death in LN patients [10,11]. Therefore, addressing nephritis may represent an effective therapeutic strategy to halt LN progression and improve survival outcomes for patients.

Dihydroartemisinin (DHA) has demonstrated various biological activities, including anti-malarial, anti-tumor, anti-viral, anti-inflammatory, and antiparasitic effects [12,13,14,15]. Research has shown that DHA can alleviate inflammatory injury in murine models of LN [16,17]. Oxymatrine, an alkaloid extracted from the radix *Sophora* genus, shares many similarities with DHA and is known for its anti-tumor, anti-inflammatory, and anti-fibrotic properties, as well as its ability to elevate white blood cell counts [18,19,20]. Additionally, oxymatrine has demonstrated therapeutic benefits in various inflammatory conditions [21,22,23], but its effects on LN have not been thoroughly investigated. Oxymatrine, recognized for its established safety profile, may contribute to inducing remission and minimizing the progression of LN to ESRD.

Yin Yang 1 (YY1), a nuclear transcription factor, has been implicated in metabolic diseases such as nonalcoholic fatty liver disease (NAFLD), obesity, and diabetic nephropathy [24,25,26]. YY1, a novel pro-inflammatory mediator, also plays a significant role in inflammation-associated diseases, including neuroinflammation, rheumatoid arthritis, and nonalcoholic hepatitis [27,28,29]. In this study, significantly elevated levels of YY1 were observed in the kidneys of LN patients.

Given the well-established role of inflammation in exacerbating LN, we investigated whether oxymatrine could relieve LN symptoms through its anti-inflammatory properties. This study aimed to explore the therapeutic potential and underlying mechanisms of oxymatrine in a murine model of LN. Findings indicate that oxymatrine exerts its therapeutic effects by inhibiting YY1 expression. These results provide strong evidence supporting the efficacy of oxymatrine and highlight YY1 as a promising therapeutic target for LN treatment. Furthermore, oxymatrine has emerged as a potential clinical candidate for LN management, likely through its inhibitory action on YY1.

## 2. Results

### 2.1. Oxymatrine Alleviates Splenomegaly, Proteinuria, and Inflammation in Mice with LN

**Figure 1:**
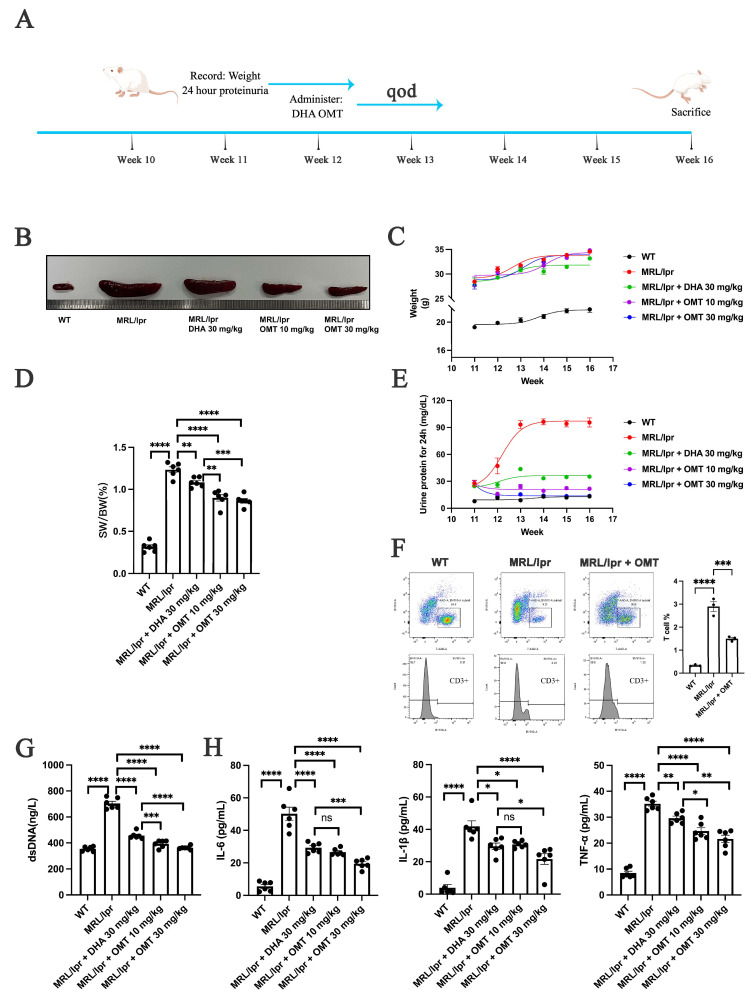
Oxymatrine alleviates splenomegaly, proteinuria, and inflammation in mice with lupus nephritis (LN). (**A**) Schematic representation of the mouse study design. (**B**) Representative images of spleens from the mice. (**C**) Weight of the mice. (**D**) Spleen weight index (total spleen weight/body weight) of the mice. (**E**) 24 h proteinuria levels in the mice. (**F**) Proportion of T lymphocyte cells. (**G**) Levels of dsDNA in the serum of the mice. (**H**) Levels of IL-6, IL-1β, and TNF-α in the serum of the mice. Each bar represents the mean ± SEM. ns, no significance, * *p* < 0.05, ** *p* < 0.01, *** *p* < 0.001, **** *p* < 0.0001; one-way ANOVA was used to compare multiple groups.

The therapeutic effects of oxymatrine on lupus nephritis (LN) symptoms were evaluated using MRL/lpr mice, with DHA serving as a positive control. Mice received oxymatrine treatment from week 12 to week 16 and were euthanized at week 16 for organ harvesting (Figure 1A). Spleen enlargement, a hallmark of LN progression in MRL/lpr mice, was significantly reduced following oxymatrine treatment compared to untreated mice. Both the spleen size and the spleen-to-body weight ratio were significantly reduced (Figure 1B–D), suggesting that oxymatrine could mitigate splenomegaly associated with LN. Proteinuria levels, which were significantly elevated in MRL/lpr mice compared to wild-type controls (WT), also showed a significant decrease following oxymatrine administration (Figure 1E). This suggests that oxymatrine may help control disease progression in mice with LN.

T lymphocytes, particularly CD3^+^ cells, play a crucial role in the pathogenesis of LN. Flow cytometric analysis showed that oxymatrine significantly reduced the proportion of CD3^+^ T cells in treated mice (Figure 1F). In addition, serum levels of dsDNA, a specific biomarker for systemic lupus erythematosus (SLE), were elevated in the MRL/lpr mice but were significantly decreased after oxymatrine treatment (Figure 1G). Moreover, inflammatory factors (IFs) were elevated in the serum of MRL/lpr mice. Oxymatrine treatment led to a significant reduction in these cytokines, indicating its potential role in alleviating systemic inflammation associated with LN (Figure 1H). In summary, these findings demonstrate that oxymatrine alleviates key symptoms of LN, including splenomegaly, proteinuria, T lymphocyte activity, dsDNA levels, and inflammation, suggesting that oxymatrine holds promise as a potential therapeutic agent for the treatment of LN.

### 2.2. Oxymatrine Attenuated the Nephritis of Mice with LN

**Figure 2:**
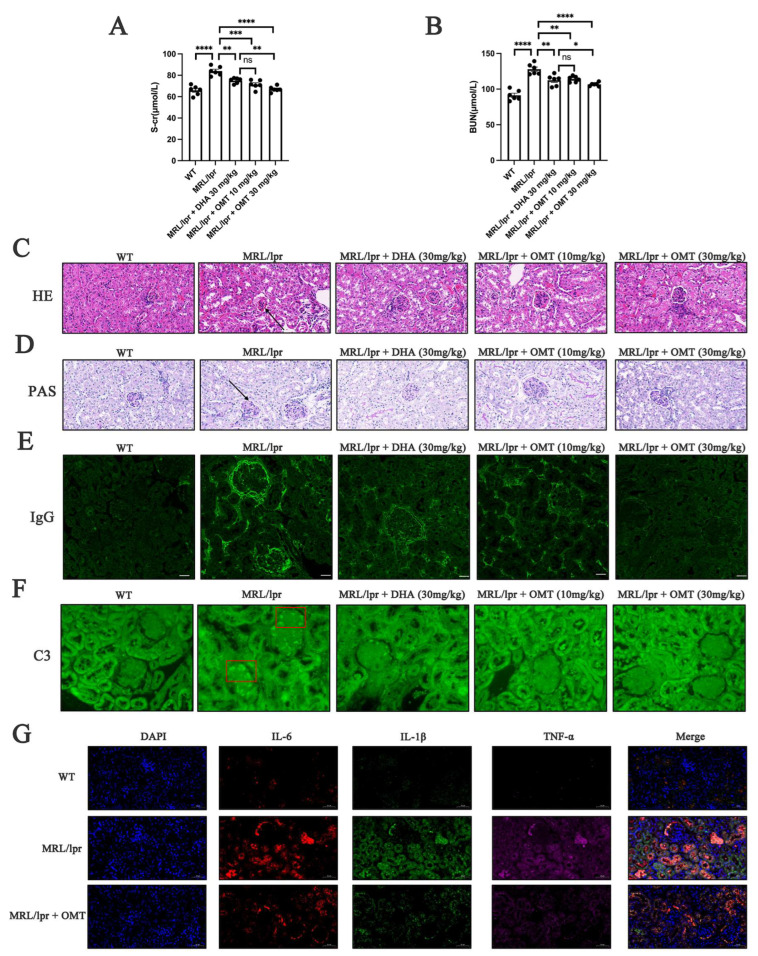
Oxymatrine improved the nephritis in mice with LN. (**A**) Serum levels of S-cr in mice. (**B**) Serum levels of BUN in mice. (**C**) Hematoxylin and eosin staining of kidney sections from the indicated groups. Scale bar = 20 μm. (**D**) Periodic Acid-Schiff staining of kidney sections from the indicated groups. Scale bar = 20 μm. (**E**) Immunoglobulin G (IgG) staining of kidney sections from the indicated groups. Scale bar = 10 μm. (**F**) Component 3 (C3) staining of kidney sections from the indicated groups. Scale bar = 10 μm. (**G**) Staining of IL-6, IL-1β, and TNF-α in kidney sections from the indicated groups. Scale bar = 20 μm. Black arrowheads indicate immune complexes, and red boxes indicate C3. Each bar represents the mean ± SEM. ns, no significance, * *p* < 0.05, ** *p* < 0.01, *** *p* < 0.001, **** *p* < 0.0001; one-way ANOVA was used to compare multiple groups.

LN is associated with compromised renal function, as demonstrated by elevated S-cr and BUN levels in mice with LN. Oxymatrine treatment significantly improved renal function in these mice, resulting in reductions in both S-cr and BUN levels compared with the MRL/lpr mice (Figure 2A,B). Furthermore, LN is characterized by the deposition of immune complexes (ICs) in renal tissues, which was significantly observed in the kidneys of untreated mice but not observed in other groups (Figure 2C,D). Immunoglobulin G (IgG) and complement component 3 (C3) deposits were significantly present around the glomeruli in the kidneys of MRL/lpr mice compared to the WT group, as shown in Figure 2E,F. However, after oxymatrine administration, the deposition of both IgG and C3 was significantly reduced, indicating a decline in immune-mediated renal damage. Furthermore, IFs were highly expressed in the renal tissues of mice with LN. Oxymatrine treatment led to a significant decrease in the production of these IFs (Figure 2G), further indicating its anti-inflammatory effects in the context of nephritis. These results demonstrate that oxymatrine effectively attenuates the nephritis symptoms in MRL/lpr mice by improving renal function, reducing IC deposition, and suppressing inflammation. These findings suggest that oxymatrine has the potential as a therapeutic agent for alleviating the renal pathology associated with LN.

### 2.3. Oxymatrine Modulates the YY1/IL-6/STAT3 Pathway to Alleviate Inflammation in LN

**Figure 3:**
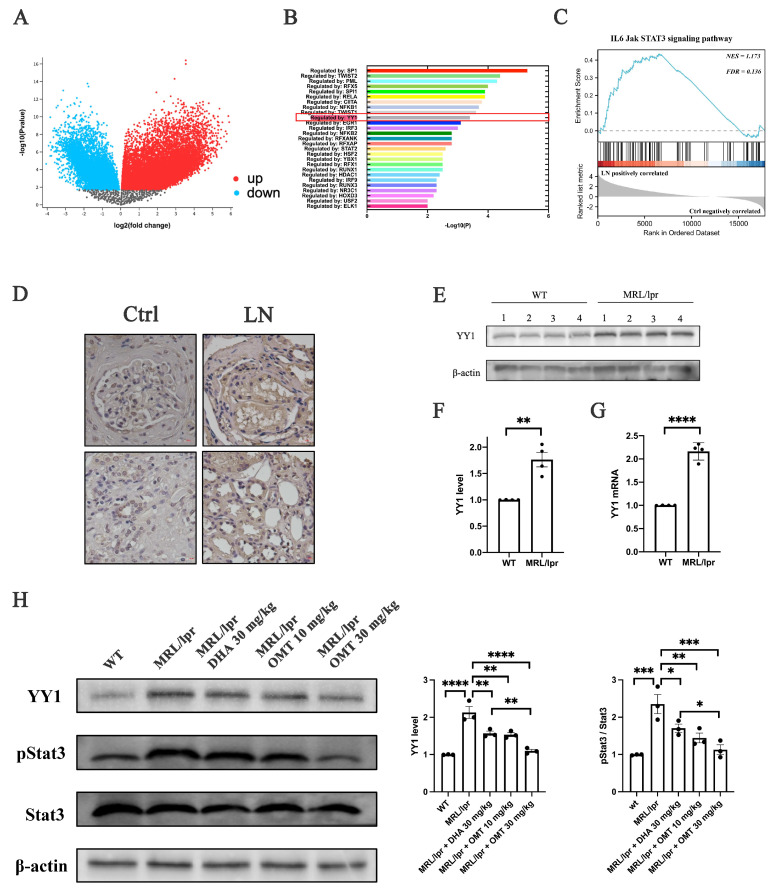
Oxymatrine modulates the YY1/IL-6/STAT3 pathway to alleviate inflammation in LN. (**A**) Volcano plots displaying differentially expressed genes (DEGs) in LN versus control (*n* = 28,439). (**B**) TRRUST analysis of DEGs from panel A. (**C**) GSEA of IL-6 Jak STAT3 signaling pathway. FDR, false discovery rate; NES, normalized enrichment score. (**D**) Relative protein levels of Yin Yang 1 (YY1) in the kidneys of LN patients assessed by IHC. Scale bar = 20 μm. (**E**,**F**) Western blot analysis of YY1 levels in the kidneys of wild-type mice and from MRL/lpr mice. The signal densities of YY1 were normalized to that of β-actin. (**G**) Quantitative real-time PCR (qRT–PCR) analysis of YY1 mRNA levels in the kidneys of wild-type mice and from MRL/lpr mice. (**H**) Western blot analysis of YY1 levels and phosphorylation levels of Stat3 in the kidneys of mice. The signal densities of YY1 were normalized to that of β-actin. Each bar represents the mean ± SEM. * *p* < 0.05, ** *p* < 0.01, *** *p* < 0.001, **** *p* < 0.0001; *t*-test and one-way ANOVA were employed to compare multiple groups.

To better understand the pathogenesis of LN and explore the potential therapeutic mechanisms of oxymatrine, previously published microarray datasets were analyzed to examine renal tissues from both control subjects and individuals affected by LN. This approach offers insights into the molecular basis of the disease and may reveal novel therapeutic targets. The gene expression profile was assessed using the microarray dataset (GSE112943) [30], sourced from human LN samples, to evaluate the clinical relevance of changes in gene expression (control group: *n* = 7, LN group: *n* = 14). Comparative genomic profiling of renal tissues from individuals with LN revealed significant differences from the profiles of control subjects (Figure 3A).

The Transcriptional Regulatory Relationships Unraveled by Sentence-based Text mining (TRRUST) analysis identified differentially expressed genes (DEGs) in the renal tissues of LN patients, which were regulated by the transcription factor Yin Yang 1 (YY1) (Figure 3B). YY1 is recognized as an inflammatory regulatory factor, and the analysis revealed that it exhibits significantly elevated expression in the renal tissues of LN patients (logFC = 2.09, adj.*P*.val = 3.39 × 10^−4^). Gene set enrichment analysis (GSEA) further revealed that the IL-6/JAK/STAT3 signaling pathway was significantly enriched for genes upregulated in LN (Figure 3C). In the renal tissues of LN patients, the expression of YY1 was significantly elevated in both renal tubules and glomeruli, as shown in Figure 3D. A similar upregulation of YY1 at both the mRNA (Figure 3G) and protein levels (Figure 3E,F) was also observed in the kidneys of mice with LN compared to control mice.

These results suggest that the pathogenesis of LN and the therapeutic potential of oxymatrine may be related to YY1. Building upon previous findings [31], the GSEA results (Figure 3C), and the observed changes in IL-6 in the renal tissues of mice with LN, it is hypothesized that oxymatrine may exert its therapeutic effects by modulating the YY1/IL-6/STAT3 signaling pathway. In the kidneys of MRL/lpr mice, a model for LN, significant increases in the expression of YY1, IL-6, and pSTAT3 were observed compared to WT mice. Oxymatrine treatment effectively reduced these expression levels, as shown in Figure 2G and Figure 3H.

These findings suggest that YY1 plays a role in the etiology of LN, and downregulating its expression could alleviate clinical symptoms. The therapeutic potential of oxymatrine is thus hypothesized to be mediated through the inhibition of the YY1/IL-6/STAT3 axis, offering a novel approach for LN treatment.

### 2.4. Oxymatrine Negatively Regulated YY1 and Played an Anti-Inflammatory Role

**Figure 4:**
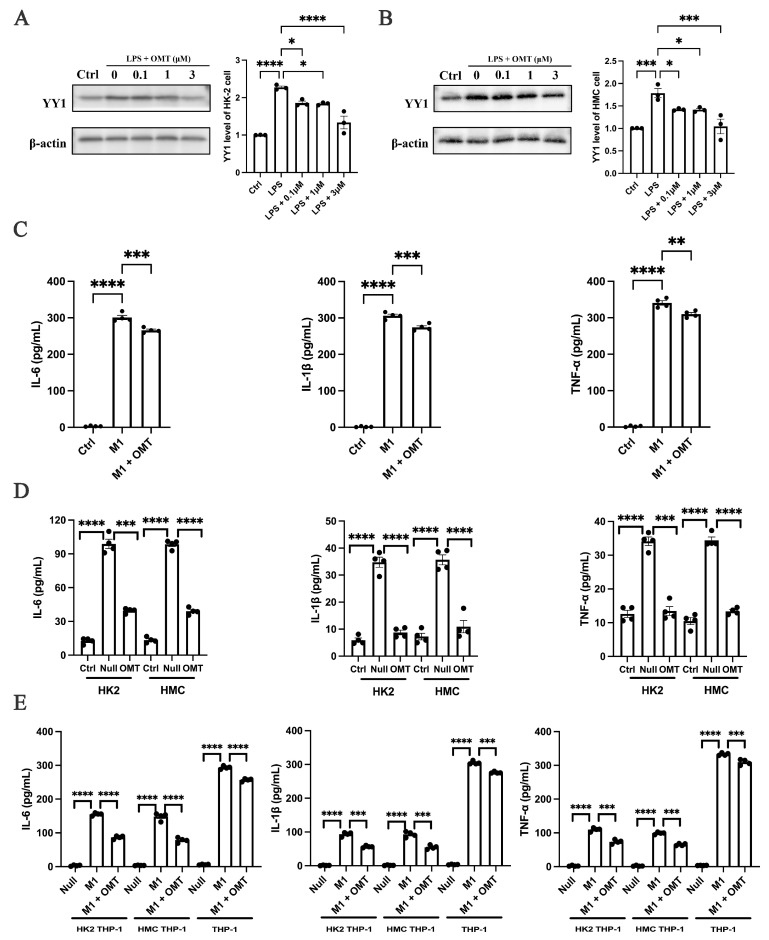
Oxymatrine inhibited the expression of YY1 and the secretion of inflammatory factors. (**A**) Western blot analysis of YY1 levels following the administration of lipopolysaccharide (LPS) and oxymatrine in HK-2 cells. The signal densities of YY1 were normalized to that of β-actin. (**B**) Western blot analysis of YY1 levels after the administration of LPS and oxymatrine in HMC cells. The signal densities of YY1 were normalized to that of β-actin. (**C**) The levels of IL-6, IL-1β, and TNF-α in the culture medium of THP-1 cells. (**D**) The levels of IL-6, IL-1β, and TNF-α in the culture medium of HK-2 and HMC cells. (**E**) The levels of IL-6, IL-1β, and TNF-α in the culture medium of HK-2, HMC, and THP-1 cells. Each bar represents the mean ± SEM. * *p* < 0.05, ** *p* < 0.01, *** *p* < 0.001, **** *p* < 0.0001; one-way ANOVA was used to compare multiple groups.

To further explore oxymatrine’s therapeutic potential in nephritis, lipopolysaccharide (LPS) was used to induce inflammation in renal parenchymal cells, specifically in the HK-2 and HMC cell lines (Figure S1). Low concentrations of oxymatrine had no significant impact on cell viability (Figure S2). Oxymatrine effectively reduced cellular inflammation by suppressing the mRNA levels of YY1 and IFs in a concentration-dependent manner (Figure S3). Furthermore, oxymatrine downregulated the expression of YY1 in a dose-dependent manner in both HK-2 (Figure 4A) and HMC cells (Figure 4B).

In the inflammatory model, M0 macrophages were stimulated to differentiate into the pro-inflammatory M1 phenotype. Oxymatrine treatment (3 μM) significantly reduced levels of IFs in M1-type macrophages (Figure 4C). Similarly, oxymatrine diminished the secretion of IFs in renal parenchymal cells (Figure 4D and Figure S3). In a co-culture model of macrophages and renal parenchymal cells, the IF secretion from parenchymal cells was significantly elevated due to the stimulatory effect of macrophages. However, oxymatrine effectively inhibited this increase (Figure 4E) and reduced the secretion of IFs in both cell types. These findings suggest that oxymatrine may suppress YY1 expression and alleviate inflammation in renal parenchymal cells. Further investigation is needed to elucidate the interactions between oxymatrine, YY1, and nephritis, potentially uncovering novel therapeutic pathways for treating this condition.

### 2.5. YY1 Promotes Inflammatory Factor Secretion in LN

**Figure 5:**
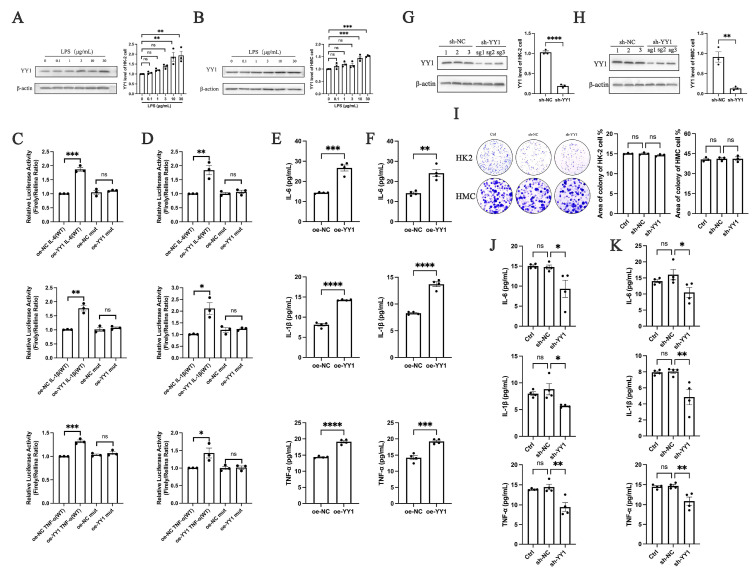
YY1 promotes inflammatory factor secretion in LN. (**A**) Western blot analysis of YY1 levels following the administration of LPS in HK-2 cells. The signal densities of YY1 were normalized to that of β-actin. (**B**) Western blot analysis of YY1 levels following the administration of LPS in HMC cells. The signal densities of YY1 were normalized to that of β-actin. (**C**) The relative activity of IL-6, IL-1β, and TNF-α in HK-2 cells was measured by luciferase assay. (**D**) The relative activity of IL-6, IL-1β, and TNF-α in HMC cells was measured by luciferase assay. (**E**) The levels of IL-6, IL-1β, and TNF-α in the culture medium of HK-2 cells after YY1 overexpression. (**F**) The levels of IL-6, IL-1β, and TNF-α in the culture medium of HMC cells after YY1 overexpression. (**G**) Western blot analysis of YY1 levels following YY1 knockdown in HK-2 cells. The signal densities of YY1 were normalized to that of β-actin. (**H**) Western blot analysis of YY1 levels following YY1 knockdown in HMC cells. The signal densities of YY1 were normalized to that of β-actin. (**I**) Cell colony formation assays of HK-2 and HMC cells after YY1 knockdown. (**J**) The levels of IL-6, IL-1β, and TNF-α in the culture medium of HK-2 cells after YY1 knockdown. (**K**) The levels of IL-6, IL-1β, and TNF-α in the culture medium of HMC cells after YY1 knockdown. Each bar represents the mean ± SEM. ns, no significance, * *p* < 0.05, ** *p* < 0.01, *** *p* < 0.001, **** *p* < 0.0001; *t*-test and one-way ANOVA were used to compare multiple groups.

The correlation between YY1 and LN requires further validation. To investigate the association between YY1 and nephritis, LPS was used to induce inflammation in renal parenchymal cells, thereby establishing a mechanistic association between YY1 expression and the inflammatory processes typical of nephritis. The experimental results demonstrated that LPS induced the expression of YY1 in a concentration-dependent manner in renal parenchymal cells, as shown in Figure 5A,B. A luciferase-based reporter assay was performed to evaluate the regulatory role of YY1 in IFs. Overexpression of YY1 in renal parenchymal cells resulted in a significant increase in the promoter activities of IL-6, IL-1β, and TNF-α compared to control groups (Figure 5C,D). At the same time, IL-6, IL-1β, and TNF-α secretion significantly increased following YY1 overexpression (Figure 5E,F). Afterward, YY1 knockdown was performed in HK-2 cells (Figure 5G) and HMC cells (Figure 5H), with no adverse effects on cell growth (Figure 5I). Compared to control groups, YY1 knockdown significantly reduced the expression levels of IL-6, IL-1β, and TNF-α (Figure 5J,K). These findings highlight the pivotal regulatory role of YY1 in modulating the secretion of IFs. Taken together, these results suggest that inhibiting YY1 can mitigate renal inflammation associated with LN. This hypothesis is further supported by the observed decrease in IF secretion following YY1 knockdown, indicating that targeting YY1 could represent a potential therapeutic strategy for managing LN.

### 2.6. Oxymatrine Improved the Symptoms of LN by Inhibiting YY1

**Figure 6:**
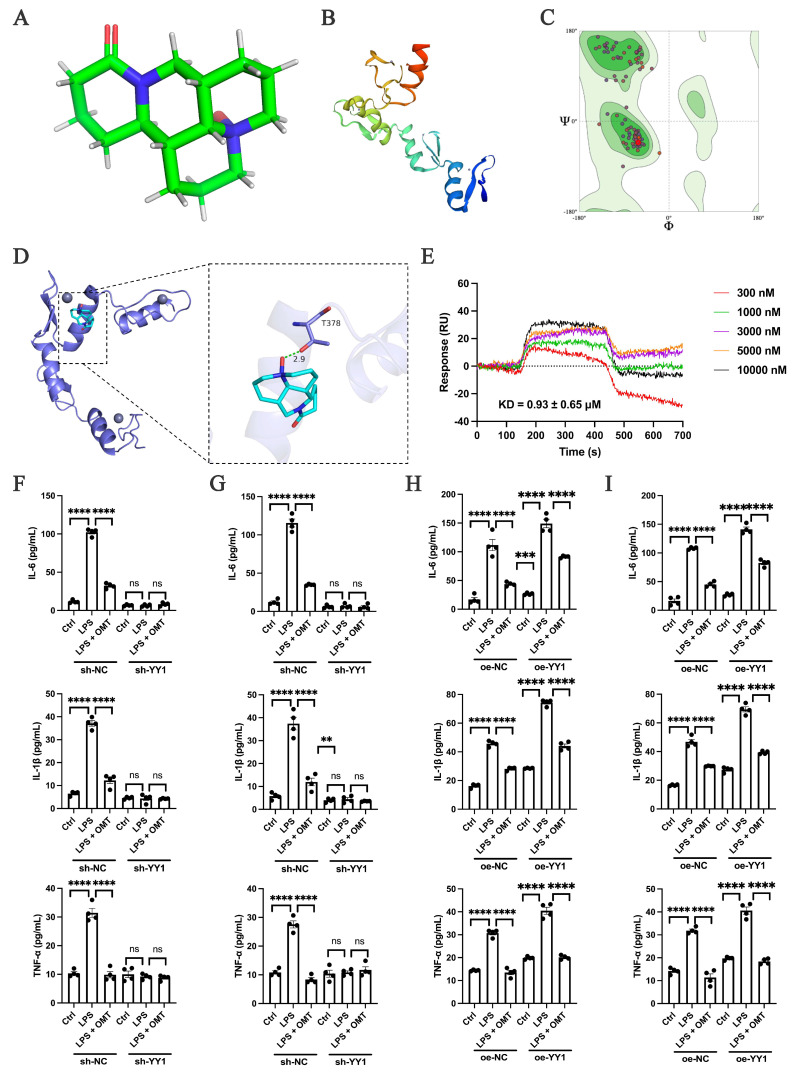
The effect of oxymatrine on LN is attributed to the inhibition of YY1. (**A**) Chemical structure of oxymatrine. (**B**) Homology modeling of YY1. (**C**) Ramachandran plots were utilized to evaluate the YY1 model. (**D**) Representative images illustrating the binding mode of oxymatrine and YY1. (**E**) Sensorgrams of oxymatrine in the SPR assay at various concentrations. (**F**) Levels of IL-6, IL-1β, and TNF-α in the culture medium of HK-2 cells following YY1 knockdown. (**G**) Levels of IL-6, IL-1β, and TNF-α in the culture medium of HMC cells following YY1 knockdown. (**H**) Levels of IL-6, IL-1β, and TNF-α in the culture medium of HK-2 cells after YY1 overexpression. (**I**) Levels of IL-6, IL-1β, and TNF-α in the culture medium of HMC cells after YY1 overexpression. Each bar represents the mean ± SEM. ns, no significance, ** *p* < 0.01, *** *p* < 0.001, **** *p* < 0.0001; one-way ANOVA was used to compare multiple groups.

The relationship between oxymatrine and YY1 requires further investigation to validate its therapeutic significance. To explore this interaction, molecular docking studies were conducted to elucidate the binding between oxymatrine (Figure 6A) and YY1 (Figure 6B). The accuracy of the constructed molecular models was assessed using Ramachandran plots (Figure 6C), a standard method for evaluating the quality of protein structure. Notably, threonine residue 378 (T378) was identified as crucial for hydrogen bond formation, significantly enhancing the binding affinity between YY1 and oxymatrine (Figure 6D). This direct binding interaction was further corroborated using surface plasmon resonance (SPR) technology, an analytical technique used to study real-time biomolecular interactions (Figure 6E). The *K_D_* for oxymatrine was calculated to be 0.93 ± 0.65 μM, indicating a direct, albeit low-affinity, binding between oxymatrine and YY1. These findings suggest that oxymatrine may modulate YY1 through direct binding, which may have therapeutic implications in diseases such as LN, where YY1 plays a significant role.

After the YY1 knockdown, there was a significant reduction in the secretion of IFs, as demonstrated in Figure 5J,K. Oxymatrine exhibited the capacity to suppress LPS-induced IFs’ secretion; however, this suppressive effect was diminished after YY1 knockdown (Figure 6F,G). In contrast, YY1 overexpression further increased IFs’ secretion (Figure 5E,F). In this context, oxymatrine continued to effectively inhibit the surge in IFs’ secretion, even after YY1 overexpression (Figure 6H,I). Given that YY1 has been identified as a critical target in LN, oxymatrine’s ability to bind to YY1 and reduce its expression suggests that it may alleviate LN symptoms by inhibiting YY1. This represents a novel therapeutic approach for the treatment of LN.

## 3. Discussion

Lupus nephritis (LN) is widely recognized as one of the most severe organ manifestations of SLE, posing a significant risk of progression to end stage renal disease (ESRD) or even mortality [2]. Previous studies [16,17] have shown that dihydroartemisinin (DHA) can alleviate the symptoms of LN, and given the similar anti-inflammatory properties of oxymatrine, we hypothesized that it could also mitigate LN symptoms. Thus, this study employed murine models to confirm oxymatrine’s efficacy, tracking changes in body weight and 24 h urine protein levels [32] starting from the 11th week. Oxymatrine was administered every other day from the 12th week, and mice were euthanized in the 16th week to assess symptoms, renal function, and pathological structures (Figure 1A). Key indicators of LN severity, such as spleen size [2] and proteinuria, were measured. The results showed significant splenomegaly in LN mice, with proteinuria levels peaking at week 14 and remaining elevated afterward. Oxymatrine treatment mitigated these changes, indicating its potential therapeutic benefit. Additionally, T lymphocytes, which are closely associated with systemic lupus erythematosus (SLE), and dsDNA, a specific marker for SLE, were reduced following oxymatrine treatment. These changes, along with reductions in inflammatory factors (IFs), further reflected the improvements in LN symptoms due to oxymatrine administration. S-cr and BUN, key indicators of renal function, were also measured. Oxymatrine partially ameliorated renal function deterioration in LN mice, suggesting its beneficial impact on kidney health. Immune complex (IC) deposition, a hallmark of LN [7], is characterized by immunoglobulin G (IgG), a type of antibody that plays a crucial role in activating the complement immune response, and component 3 (C3), the most abundant component of the complement system [33,34]. LN mice showed significant accumulation of IgG and C3 around the glomeruli. After treatment with oxymatrine, this deposition was significantly reduced. These findings suggest that oxymatrine may be a promising compound that can potentially alleviate the symptoms of LN.

Research into renal changes in LN patients is essential for understanding the disease’s pathogenesis and oxymatrine’s therapeutic mechanisms. This study’s comparative analysis of upregulated genes in LN patient kidneys versus controls identified Yin Yang 1 (YY1) as a major regulator of these genes. Multiple studies [27,28] have confirmed that YY1 is a pro-inflammatory mediator, with elevated expression in both LN patients and MRL/lpr mice, similar to IL-6. Our findings indicate that oxymatrine exerts its effects by inhibiting the YY1/IL-6/STAT3 signaling pathway, providing insight into its mechanism of action. However, further investigation is needed to fully understand YY1’s role in LN progression and its modulation by oxymatrine.

Renal tubular epithelial cells (RTECs) and glomerular mesangial cells (GMCs), which comprise most renal parenchymal cells, are critical sites of chronic inflammation that lead to nephron structural damage and progressive renal dysfunction [30,35]. Targeting this inflammation is a key strategy for exploring LN therapeutic mechanisms. In this study, inflammation was induced in cellular models using lipopolysaccharide (LPS) at a concentration of 10 μg/mL, which upregulated YY1 expression [27]. Oxymatrine, at 3 μM, effectively inhibited YY1 expression and reduced the release of inflammatory factors, thereby reducing the inflammatory response in renal parenchymal cells. Studies demonstrated the regulatory role of YY1 in the secretion of IFs [36,37,38]. Findings indicate that inhibiting YY1 reduces IFs’ secretion, potentially alleviating LN symptoms [39]. To explore this, the interaction between oxymatrine and YY1 was investigated using molecular docking and SPR analyses, suggesting that oxymatrine inhibits YY1 activity. However, the effects observed were modest, and it is important to note that molecular docking is a predictive simulation tool. Further investigation was conducted to explore the mechanism of oxymatrine’s inhibition of YY1. Experiments involving the knockdown and overexpression of YY1 in cellular models were performed, followed by the administration of oxymatrine to the respective groups. The knockdown of YY1 made the therapeutic intervention with oxymatrine ineffective, while its overexpression did not reduce oxymatrine’s effects. These findings suggest that oxymatrine alleviates LN symptoms by directly suppressing YY1 expression.

This study demonstrated that oxymatrine exerts its therapeutic effects on LN by inhibiting YY1 expression and suppressing the YY1/IL-6/STAT3 signaling pathway. It also highlights the essential role of YY1 in the protective effects of oxymatrine against LN. Drug screening targeting YY1 may provide further insights into the pathogenesis of LN. However, certain limitations of this study need to be acknowledged. Initially, a preliminary validation of the molecular docking results was carried out using SPR experiments; however, further experimental confirmation was lacking. The weak affinity between oxymatrine and YY1 suggests the potential existence of other inhibitors with an improved affinity that may result in optimum therapeutic effects. Oxymatrine exhibits multifaceted anti-inflammatory properties by inhibiting the suppressor of cytokine signaling 1 (SOCS1) [21] and nuclear factor kappa-light-chain-enhancer of activated B cells (NF-κB) [22]. Oxymatrine may also exert its pharmacological effects for the treatment of LN via these targets. Further research is needed to explore other potential targets of action for oxymatrine. Similarly, LN is a chronic disease, and there is a lack of long-term studies to evaluate the lasting effects of oxymatrine, with limited exploration of potential side effects.

## 4. Materials and Methods

### 4.1. Patients

The study was approved by the Ethics Committee of Jinan University First Affiliated Hospital (approval number: KYk-2022-013). Clinical and renal histopathological data were collected from patients with LN, which was confirmed through renal biopsies. All patients met the 2003 American College of Rheumatology revised criteria for SLE and were diagnosed with LN based on renal biopsy results. Clinical and kidney histopathological data of patients with renal biopsies proven LN diagnosis were collected (*n* = 8 patients, of whom 2 had class III LN, 4 had class IV LN, and 2 had class IV plus class V LN). Clinical information of patients with lupus nephritis (renal biopsies) is given in Table S1.

### 4.2. Animals

All experimental procedures adhered to guidelines established by the Institutional Animal Care and Use Committee of Southern Medical University (approval number: SMUL2023014). Mice were housed in a controlled environment with a temperature (24 ± 1 °C) and a 12:12 h light/dark cycle, with access to food and water ad libitum. Female C57BL/6 background MRL/lpr mice (10 weeks old, weighing 20–30 g) were obtained from Shanghai Lingchang Biotechnology Co., Ltd. (Shanghai, China). MRL/lpr mice were divided into vehicle control, oxymatrine low dose group (10 mg/kg), oxymatrine high dose group (30 mg/kg), and DHA group (30 mg/kg) (*n* = 6 for each group) [40]. C57/BL6 served as normal control (WT) in animal experiments. DHA (MedChemExpress, MCE, Monmouth Junction, NJ, USA) as positive control was administered at a concentration determined by previous studies [16,17], and all mice received the drug (oxymatrine) via intraperitoneal injection every other day.

### 4.3. Cell Culture

The human proximal tubular epithelial cell line (HK-2), human glomerular mesangial cell line (HMC), and human monocytic leukemia cell line (THP-1) were obtained from the American Type Culture Collection (ATCC, Manassas, VA, USA). All cell lines were cultured in either Dulbecco’s Modified Eagle Medium (DMEM, Gibco, Waltham, MA, USA) or RPMI 1640 (Gibco, USA), supplemented with 10% fetal bovine serum (FBS, Gibco, USA), 100 U/mL penicillin (Gibco, USA), and 100 μg/mL streptomycin (Gibco, USA). The cultures were maintained at 37 °C in a 5% CO_2_ humidified incubator. M0-type macrophages were produced by treating THP-1 cells with phorbol 12-myristate 13-acetate (100 ng/mL) (PMA, MCE, USA) for 24 h. M1-type macrophages were produced by treating M0-type macrophages with lipopolysaccharide (LPS, MCE, USA) (100 ng/mL) and IFN-γ (20 ng/mL) (Thermo Fisher Scientific, Waltham, MA, USA) for an additional 24 h. Oxymatrine (MCE, USA) was added to the culture medium 12 h after induction and allowed to act for another 24 h.

### 4.4. Enzyme-Linked Immunosorbent Assay (ELISA)

Serum samples and cell culture supernatants were analyzed to assess the levels of IFs. Commercial ELISA kits (Biolegend, San Diego, CA, USA) were used to quantify inflammatory cytokines, including IL-1β, IL-6, and TNF-α, in both mouse serum and cell culture supernatants. Additionally, double-stranded DNA (dsDNA), blood urea nitrogen (BUN), and serum creatinine (S-cr) levels were measured using mouse-specific ELISA kits (Meimian, Shanghai, China). Cytokine concentrations were determined based on standard curves.

### 4.5. Flow Cytometric Analysis of Cells

Single-cell suspensions were prepared from harvested spleens, and immunophenotyping was conducted using the anti-CD3-BV510 fluorescent antibody (Biolegend, USA). Flow cytometry was performed using the LSRFortessa^TM^ X-20 cell analyzer (BD Biosciences, San Jose, CA, USA).

### 4.6. Histology Analysis

Kidney samples were fixed in 4% paraformaldehyde and embedded in paraffin. Sections (4 μm thick) were cut perpendicularly to the long axis of the kidney for immunohistochemistry and morphometric analysis. Hematoxylin and eosin (H&E) staining and periodic acid-Schiff (PAS) staining were performed to assess kidney morphology and immune complex deposition. Staining protocols have been previously described [41]. The sections were examined using an Olympus BX43F microscope (Olympus, Tokyo, Japan).

### 4.7. Immunofluorescence Analysis

Immunohistochemistry methods were conducted as described previously [42]. Paraffin-embedded kidney sections were deparaffinized in xylene, hydrated in graded alcohol and water, and treated with 3% H_2_O_2_ to block endogenous peroxidase activity. The sections were blocked with normal goat serum and incubated overnight at 4 °C with primary antibodies: IgG (1:300, HUABIO, Hangzhou, China), C3 (1:300, HUABIO, China), IL-6 (1:300, Abcam, Cambridge, UK), IL-1β (1:300, Abcam, UK), and TNF-α (1:300, Abcam, UK). Endogenous peroxidase blockers were applied prior to incubation with the corresponding HRP-conjugated secondary antibodies (Beyotime, Nantong, China). The samples were visualized using an Olympus BX43 microscope.

### 4.8. Data Acquisition

Gene expression profiles (GSE112943) were retrieved from the NCBI Gene Expression Omnibus (GEO, https://www.ncbi.nlm.nih.gov/geo/ (1 November 2023)). Kidney gene expression profiles consisting of 7 normal samples and 14 LN samples were extracted from the database. The data were visualized using a volcano plot generated by GEO2R, with filter criteria set at logFC > 2 and *p* < 0.05. For enrichment analysis, differentially expressed genes (DEGs) were analyzed using Metascape (https://metascape.org/ (13 November 2023)) to identify seed genes based on Gene Ontology (GO) terms and Kyoto Encyclopedia of Genes and Genomes (KEGG) pathway analysis.

### 4.9. Immunohistochemistry Analysis

Paraffin-embedded kidney sections were deparaffinized in xylene, followed by hydration in graded alcohol and water. The sections were treated with 3% hydrogen peroxide (H_2_O_2_) to block endogenous peroxidase activity. After blocking with normal goat serum, the sections were incubated overnight at 4 °C with a primary antibody against YY1 (1:300, Protein-tech, Rosemont, IL, USA). A polymer HRP detection system was used for visualization, and the sections were counterstained with hematoxylin. The stained sections were examined under an Olympus BX43 microscope.

### 4.10. Western Blot Analysis

Total proteins were extracted from renal cortical tissues using a lysis buffer (Beyotime, China). Proteins were separated via 10% SDS-PAGE and transferred to polyvinylidene difluoride (PVDF) membranes (Millipore, Burlington, MA, USA), following established procedures [43]. The membranes were incubated overnight at 4 °C with primary antibodies, including anti-YY1, anti-p-Stat3 (Cell signaling Technology, CST, Danvers, MA, USA), anti-Stat3 (CST, USA), and anti-β-actin (CST, USA) (all at 1:1000). Then, secondary antibodies (1:3000 dilution) were applied for 1 h at room temperature. Detection was performed using a LI-COR Odyssey infrared scanner (LI-COR BIO, Lincoln, NE, USA), and band intensities were analyzed with ImageJ version 13.0.6 software (National Institutes of Health, Bethesda, MD, USA).

### 4.11. Knockdown and Overexpression

Lentiviral vectors targeting YY1 (shYY1) and nontargeting controls (shNC) were synthesized by GenePharma (Shanghai, China). Plasmids for YY1 overexpression (pCMV-Flag-YY1) and the negative control (pCMV-Flag-NC) were constructed in-house. All transfections were performed using Lipofectamine 3000 (Thermo Fisher Scientific, USA) according to the manufacturer’s protocol. The sequences of primers are provided in Table S2.

### 4.12. Luciferase Assay

Luciferase assays were conducted as described previously [31]. Cells at 30% confluence were co-transfected using PolyJet with a mixture containing the IL-6, IL-1β, or TNF-α-dependent luciferase reporter, along with YY1 overexpression vector and Renilla luciferase. After 48 h, the cells were lysed, and luciferase activity was measured using a dual-luciferase reporter assay system (Promega, Madison, WI, USA), according to the manufacturer’s protocol.

### 4.13. Colony Formation Assay

Cells were seeded at a density of 800 cells per well in 6-well plates and incubated at 37 °C in a 5% CO_2_ humidified incubator for 10 days. After incubation, colonies were washed with cold PBS, fixed in methanol, and stained with 0.5% crystal violet (Solarbio, Beijing, China). Colony formation was quantified by counting the number of stained colonies.

### 4.14. Molecular Docking

The amino acid sequence of human YY1 (NP_003394.1) was retrieved from the NCBI database (https://www.ncbi.nlm.nih.gov/gene/ (1 February 2024)). Homology modeling of YY1 was performed using SWISS-MODEL (https://swissmodel.expasy.org (1 February 2024)), and model quality was evaluated using a Ramachandran plot. Oxymatrine’s molecular structure was generated in ChemDraw version 20.0 and converted to SDF format. Homology models of YY1 were prepared using the Surflex-Dock module. Molecular docking experiments between the processed receptor, YY1, and energy-optimized ligand, oxymatrine, were conducted using Discovery Studio 3.0. Docking was performed in standard precision (SP) mode, allowing the ligand to move freely while keeping the receptor rigid. Glide SP scores were used to identify potential hits for further analysis. The docking results were visualized using PyMOL version 3.1 software.

### 4.15. Surface Plasmon Resonance for Affinity Screening and Affinity Determination

A PlexArray HT A100 optical biosensor was used for affinity screening and to determine the equilibrium dissociation constant (*K_D_*) for protein–ligand interactions. The chip channel was activated with 1-ethyl-3-(3-dimethylaminopropyl) carbodiimide (EDC; GE Healthcare, Chicago, IL, USA) and N-hydroxysuccinimide (NHS; GE Healthcare) before the YY1 protein was coupled to the chip. Various concentrations of small molecule compounds were introduced, from low to high concentrations, and the interaction kinetics were recorded in real time. Molecular weight adjustments and solvent corrections were applied to eliminate non-specific binding and signal drift effects. Data processing was performed using PlexArray HT A100 analysis software (Plexera, Woodinville, WA, USA).

### 4.16. Statistical Analysis

Statistical analyses were performed using GraphPad Prism 9.0 software (San Diego, CA, USA). All data are expressed as mean ± standard error of the mean (SEM). Each experiment was conducted at least three times. Differences between groups were evaluated using a Student’s *t*-test or one-way ANOVA followed by Tukey’s post hoc test, where appropriate. A *p*-value < 0.05 was considered statistically significant.

## 5. Conclusions

This study provides novel insights into the pathogenesis of LN and identifies potential therapeutic strategies. Both in vitro (cell culture) and in vivo (animal model) investigations have demonstrated the critical role of the YY1 transcription factor in mediating the protective effects of oxymatrine. By inhibiting YY1, oxymatrine has demonstrated potential as a therapeutic agent, offering a promising approach to mitigating symptoms and improving disease outcomes in LN. These findings position oxymatrine as a viable candidate for further exploration in treating LN, particularly through its modulation of the YY1/IL-6/STAT3 signaling pathway.

## Data Availability

All data analyzed during this study are included in this published article and its Supplementary Files. The detailed experimental procedures and the materials will be freely available to the scientific community upon reasonable request.

## Supplementary Materials

The supporting information can be downloaded at https://www.mdpi.com/article/10.3390/ijms252212260/s1.
