# Supplementary material for: Oxymatrine Ameliorates Lupus Nephritis by Targeting the YY1-Mediated IL-6/STAT3 Axis
Source: Int J Mol Sci. 2024 Nov 14;25(22):12260. doi: 10.3390/ijms252212260 (PMC11594375; doi:10.3390/ijms252212260)
Supplement: Supplementary file 1 [file ijms-25-12260-s001.zip › ijms-3286423-supplementary.pdf]

Supplemental Data

Oxymatrine Ameliorates Lupus Nephritis by Targeting the YY1-Mediated IL-6/STAT3 Axis

Haoxing Yuan [1,†], Zheng Peng [1,†], Honglian Li [1], Yuzhen Rao [1], Kunyu Lu [1], Chan Yang [1], Chen Cheng [1] and Shuwen Liu [1,2,3,*]

[1] Guangdong Provincial Key Laboratory of New Drug Screening, NMPA Key Laboratory of Drug Metabolism Research and Evaluation, School of Pharmaceutical Sciences, Southern Medical University, Guangzhou 510515, China; yuanhaoxing1222@163.com (H.Y.); pengzheng2014@163.com (Z.P.); lihonglian202101@163.com (H.L.); yzraohorris@gmail.com (Y.R.); lky886@smu.edu.cn (K.L.); virus6522@smu.edu.cn (C.Y.); chengchen1997@i.smu.edu.cn (C.C.)

[2] State Key Laboratory of Organ Failure Research, Guangdong Provincial Institute of Nephrology, Southern Medical University, Guangzhou 510515, China

[3] Innovation Center for Medical Basic Research on Inflammation and Immune Related Diseases, Ministry of Education, Southern Medical University, Guangzhou 510515, China

* Correspondence: liusw@smu.edu.cn

† These authors contributed equally to this work.

1. Supplementary Materials

Oxymatrine (HY-N0158), dihydroartemisinin (HY-N0176), lipopolysaccharides (LPS, HY-D1056), and Phorbol 12-myristate 13-acetate (PMA, HY-18739) were from MedChemExpress (Shanghai, China). Recombinant Human IFN-γ was from Thermo Fisher Scientific (MA, USA). The primary antibody for YY1 (22156-1-AP) was from Proteintech (IL, USA), and the primary antibodies for β-actin (4970), pStat3 (9145), and Stat3 (9139) were from Cell Signaling Technology (MA, USA). Secondary antibodies used were HRP-labeled Goat Anti-Rabbit IgG(H+L) (Beyotime, A0208) and HRP-labeled Goat Anti-Mouse IgG(H+L) (Beyotime, A0216). IL-6 (430504), IL-1β (437004), and TNF-α (430204) ELISA Kit for Human, as well as IL-6 (431304), IL-1β (432604) and TNF-α (430904) ELISA Kit for Mouse were from Biolegend (CA, USA); dsDNA-Ab (MM-45766M1), BUN (MM-0692M1) and S-cr (MM-44455M1) ELISA Kit were from MeiMian (Shanghai, China). Dimethyl sulfoxide (DMSO) was from Sigma (Shanghai, China). RIPA lysis buffer, protease, and phosphatase inhibitor cocktail were from Beyotime (Nanjing, China). Crystal Violet Stain solution (G1062) was from Solarbio. Rabbit Anti-Mouse IgG antibody (HA1030) and Rabbit Anti-C3 Antibody (JF10-30) were from HUABIO (Hangzhou, China). Anti-mouse CD3-BV510 antibody (17A2) was from Biolegend (CA, USA). CY3 (HY-D0822), FITC (HY-66019), and CY5 (HY-D0821) were from MedChemExpress (Shanghai, China). Rabbit Anti-Goat IL-6 antibody (ab290735), Rabbit Anti-Goat IL-1β antibody (ab254360), and Rabbit Anti-Goat TNF-α antibody (ab183218) were from Abcam (Lon, UK). The dual-luciferase reporter assay system (E1910) was from Promega (Madison, USA). All the primers used are listed in Table S1.

2. Supplementary Methods

2.1. Quantitative RT-PCR

Total mRNA was extracted using TRIzol reagent (Invitrogen, China). Complementary DNA was synthesized using PrimeScript RT Reagent Kit with gDNA Eraser (TaKaRa, China). RT-PCR was performed by SYBR Premix Ex TaqTM II (TaKaRa, China). GAPDH was used as an endogenous control.

2.2. Cell proliferation Assay

Cells were seeded in a 96-well plate at a density of 4×10^3 cells/100 µL per well and incubated overnight. They were treated with oxymatrine or DMSO overnight. CCK8 reagent (10 µL) (meilunbio, China) was added to each well. Cells were then incubated at 37 ℃ in the dark for 1 hour. The absorbance was measured at 450 nm using a spectrophotometer.

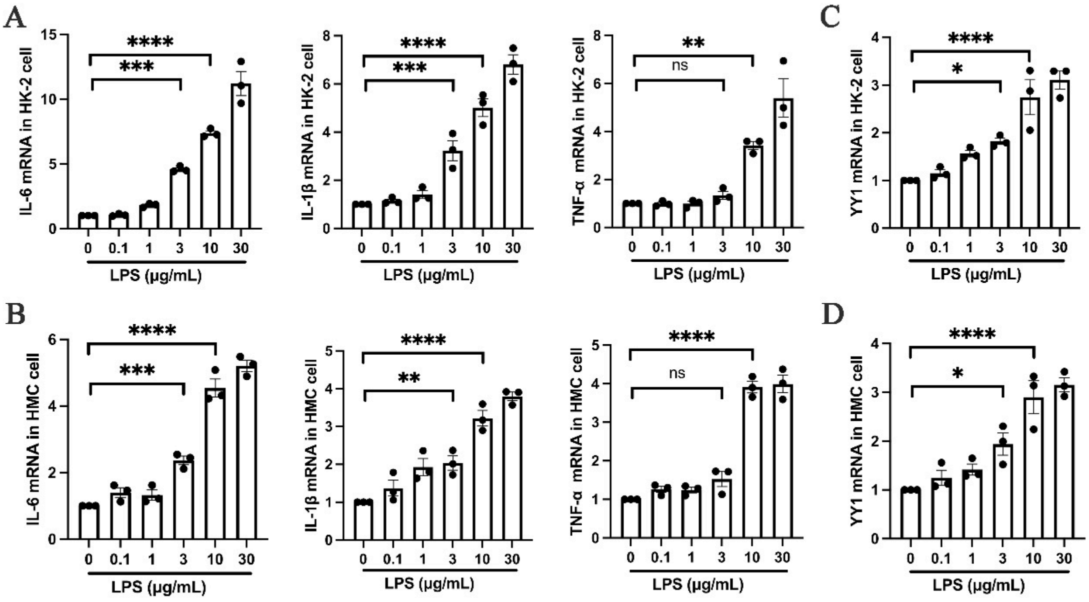

Figure S1. LPS induced the secretion of YY1 and inflammatory factor. (**A**) Quantitative real-time PCR (qRT–PCR) analysis of IL-6, IL-1β and TNF-α mRNA levels in HK-2 cells. (**B**) qRT–PCR analysis of IL-6, IL-1β, and TNF-α mRNA levels in HMC cells. (**C**) qRT–PCR analysis of YY1 mRNA levels in HK-2 cells. (**D**) qRT–PCR analysis of YY1 mRNA levels in HMC cells. Each bar represents the mean ± SEM. ns, no significance, $^{*}P < 0.05$, $^{**}P < 0.01$, $^{***}P < 0.001$, $^{****}P < 0.0001$, one-way ANOVA was used to compare multiple groups.

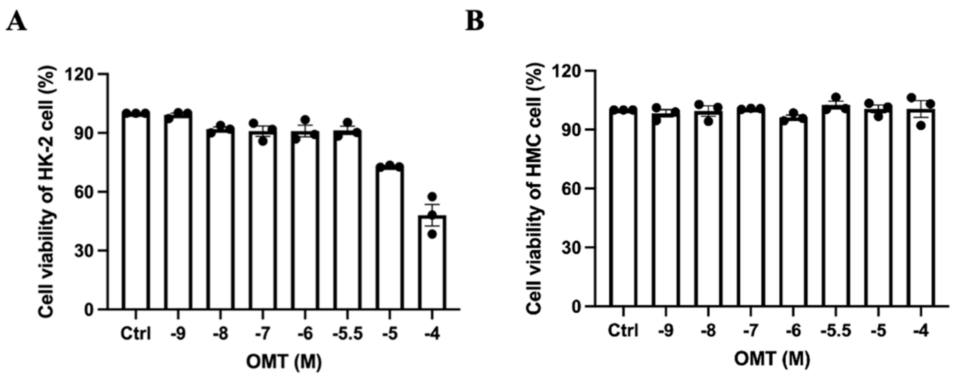

Figure S2. Dose-response curves of cells following oxymatrine treatment. (**A**) Cell viability of HK-2 cells after administering oxymatrine. (**B**) Cell viability of HMC cells after administering oxymatrine.

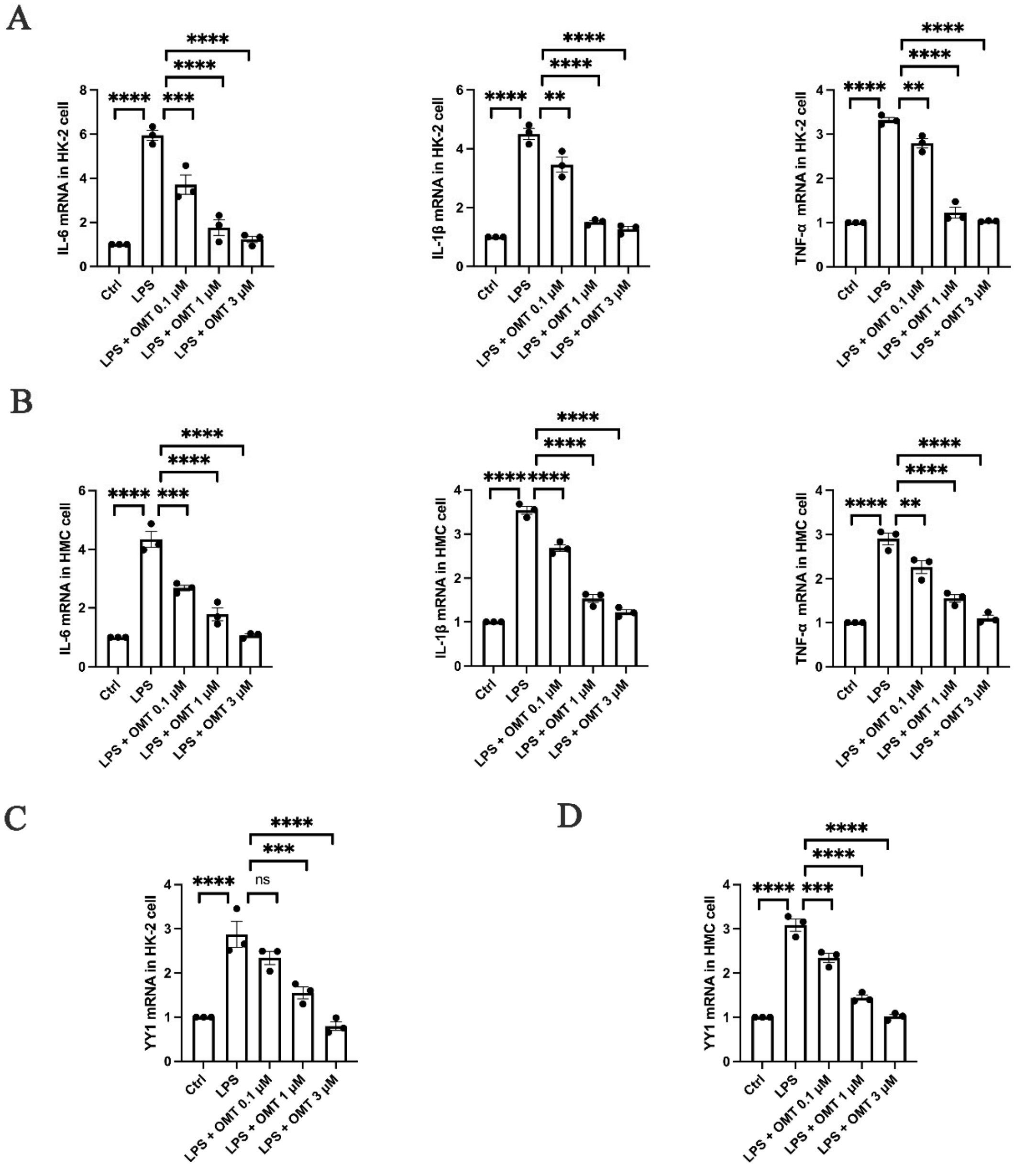

Figure S3. Oxymatrine inhibited the expression of YY1 and secretion of inflammatory factor. (**A**) Quantitative real-time PCR (qRT–PCR) analysis of IL-6, IL-1β, and TNF-α mRNA levels in HK-2 cells. (**B**) qRT–PCR analysis of IL-6, IL-1β, and TNF-α mRNA levels in HMC cells. (**C**) qRT–PCR analysis of YY1 mRNA levels in HK-2 cells. (**D**) qRT–PCR analysis of YY1 mRNA levels in HMC cells. Each bar represents the mean ± SEM. ns, no significance, $^{*}P < 0.05$, $^{**}P < 0.01$, $^{***}P < 0.001$, $^{****}P < 0.000$, one-way ANOVA was used to compare multiple groups.

Table S1. Information of lupus nephritis patients.

Patients	Age	Gender	Anti-dsDNA-Ab (IU/ml)	Anti-nuclear-Ab (titer)	Anti-Sm-Ab	Urinary microalbumin (mg/L)	LN (Stage/Class)
1	34	Female	178	160	(-)	235	III
2	32	Female	＞300	160	(-)	140	IV
3	30	Female	＞300	＞500	(-)	258	IV plus V
4	36	Female	155	＞500	(+)	1208	III
5	27	Female	41	316	(-)	910	IV
6	21	Female	118	227	(-)	1198	IV plus V
7	44	Female	＞300	422	(+)	2506	IV
8	20	Female	261	316	(-)	1976	IV

Table S2. Sequences of primers used in this study

		**Primers**
GAPDH	Forward	GAACGGGAAGCTCACTGG
	Reverse	GCCTGCTTCACCACCTTCT
YY1	Forward	CTGGCATTGACCTCTCAGATC
	Reverse	GCCGAGTTATCCCTGAACATC
IL-6	Forward	CAATGAAAAGGCCCTCTAGT
	Reverse	GAGGGAGAAGTTTTGCCTAA
IL-1β	Forward	TGAAGAAAGAAGGGGGTCTT
	Reverse	TCAACAAAATGACCCTGCTA
TNF-α	Forward	TGCTCACTAAGTGTGTATGG
	Reverse	TATTCACCTTCCAGGCATTC
IL-6 promoter	P1	TTTCTCTATCGATAGGTACCGGTCCTTGATGTAACAGCCAGGATCAAACAG
	P2	CTTAGATCGCAGATCTCGAGAGCTGGGCTCCTGGAGGGGAGATAGAGC
IL-1β promoter	P1	TTTCTCTATCGATAGGTACCCACGATGCACCTGTACGATCACTGAA
	P2	CTTAGATCGCAGATCTCGAGGAAGACACAAATTGCATGGTGAAGTCAG
TNF-α promoter	P1	TTTCTCTATCGATAGGTACCAGCAGACGCTCCCTCAGCAAGGAC
	P2	CTTAGATCGCAGATCTCGAGCTCGCCACTGAATAGTAGGGCGATT
oeYY1	P1	CACACTGGACTAGTGGATCCCGCCACCATGGCCTCGGGCGACACCCTC
	P2	CCTTGTAGTCACTTAAGCTCTGGTTGTTTTTGGCCTTAGCATG
shYY1		AGCCGTCCGTGGCGATGTAGG
shNC		GTTCTCCGAACGTGTCACGTT
